# Coping strategies and quality of life: a longitudinal study of high-grade glioma patient-caregiver dyads

**DOI:** 10.1186/s12955-018-0983-y

**Published:** 2018-08-02

**Authors:** Karine Baumstarck, Olivier Chinot, Emeline Tabouret, Patrizia Farina, Marilyne Barrié, Chantal Campello, Gregorio Petrirena, Zeinab Hamidou, Pascal Auquier

**Affiliations:** 1 EA 3279 CEReSS - Health Service Research and Quality of Life Center, Aix Marseille Université, School of medicine - La Timone Medical Campus, 27 bd Jean Moulin, F-13385 Marseille, cedex 05 France; 2 National Clinical research Quality of Life in Oncology Platform, Marseille, France; 3 Department of Neuro-Oncology, Assistance Publique Hôpitaux de Marseille, Timone Hospital, 13005 Marseille, France

**Keywords:** High-grade gliomas, Caregivers, Dyads, Quality of life, Coping, Social support

## Abstract

**Background:**

Among a sample of patient-informal caregiver dyads in the specific context of new diagnoses of high-grade glioma in the time-frame between diagnosis and the third month following diagnosis, we examine whether the coping strategies implemented by the patients and their caregivers influenced their own quality of life (QoL) and the QoL of their relatives.

**Methods:**

Thirty-eight dyads with patients having recent diagnoses of high-grade glioma were involved in this longitudinal study. The self-reported data include QoL (Patient-Generated Index, EORTC QLQ-C30, and CareGiver Oncology Quality of Life), and coping strategies (BriefCope). Data were collected at T1 corresponding to the time-frame between diagnosis and postsurgical treatment initiation and T2 corresponding to the 3-month post-inclusion follow-up.

**Results:**

Coping strategies based on social support and avoidance were the least used at baseline and the 3-month follow-up, both for patients and caregivers. At the 3-month follow-up, the use of social support at baseline was significantly related to lower scores of QoL for the patients and with higher QoL for the caregivers. For the patient, the use of problem-solving or positive thinking at baseline was not related to his/her QoL, while it was related to more satisfactory QoL scores for the caregiver. The use of avoidance at baseline was linked to a higher 3-month QoL for the patients and a lower 3-month QoL for the caregivers. Using the specific dyadic analyses (actor–partner interdependence model), the 3-month patient’s QoL was lower (β = − 0.322; *p* = 0.03) when the patient mobilized the social support strategy at baseline, but was higher(β = 0.631; *p* < 10^− 3^) when his/her informal caregiver used this strategy. After adjustment for sex, age, and baseline PGI score, the link between high use of the social support strategy at baseline by the caregiver and the patient’s 3-month QoL, remained present (positive partner effect; β =0.675; *p* < 10^− 3^).

**Conclusion:**

The QoL for patients and their informal caregivers since the time of diagnosis is directly related to the use of coping strategies based on social support at time of diagnosis.

## Background

The diagnosis of a high-grade glioma diagnosis causes major lifestyle disruptions for both patients and their relatives. These disruptions have considerable social, emotional, psychological and physical consequences [1–3], leading to an significant quality of life (QoL) alteration [4–10].

Coping is commonly defined as the cognitive and behavioral efforts that are implemented to solve problems and reduce the stress that these problems may cause [11, 12]. Several coping strategies can be used in stressful situations [13]. The personal ability to cope has been shown to directly impact on the QoL of individuals. The nature of an individual’s coping strategies may directly impact not only their own QoL, but also the QoL of the family caregiver. Previous studies have examined these effects in various contexts, such as when individuals have cancer [14, 15], severe mental diseases [16], or hearing impairment [17]. Coping strategies based on problem-solving or positive thinking appear to be associated with a better QoL, while coping strategies based on avoidance or social support appear to be a psychological risk factor for a lower QoL [18]. However, all such studies used observational and cross-sectional designs, which do not allow for causality inferences to be made between coping strategies and QoL.

Patients with high-grade gliomas and their family caregivers confront a disease characterized by a potentially short terminal trajectory and severe functional cognitive and neuropsychological sequelae that cause major lifestyle disruptions. The events immediately following a diagnosis impact specific domains in the life of the patient that differ from the impacted domains in the life of the family caregiver. Because of the rapid progression of the disease, patients and their family caregivers have a little time to adapt and must quickly develop specific coping strategies. For these reasons, there is an interest in studying the mechanisms of the interconnections within the patient-caregiver dyad in the specific context of the recent announcement of a high-grade glioma diagnosis. At present, it remains unknown whether an individual’s coping strategies actually influence their QoL and that of his/her relatives over time.

Our sample includes patient-caregiver dyads in the specific context of new diagnoses of high-grade glioma in the time-frame between diagnosis and the first three months following diagnosis. In this sample, we examine whether the coping strategies implemented by the patients and their caregivers at the time of diagnosis influenced their patients’ QoL and the QoL of their relatives. This study used the actor–partner interdependence model (APIM) [19].

## Methods

### Design and settings

We conducted a longitudinal study. The recruitment of patient-caregiver dyads was made in the Neuro-oncology Department of the public Timone Hospital through the regional glioma cohort implemented near Marseille in the South of France. This cohort is part of the French “Site de Recherche Intégrée sur le Cancer (SIRIC) gliomas program”, which is a research program that is certified by the French National Cancer Institute, at which all clinical teams work in the field of gliomas to form a better understanding of the pathology and to better identify efficient therapeutic approaches and improved care for patients suffering from gliomas (http://fr.ap-hm.fr/cancerologie/recherche-et-essais-cliniques/siric-site-de-recherche-en-cancerologie). In this study, we reported the data collected at the first two assessments: T1, corresponding to the time between diagnosis and postsurgical treatment initiation, and T2, corresponding to the 3-month post-inclusion follow-up. The rationale of the T2 time point relies on the first tumoral progression assessment (3 months post treatment).

### Sample selection

The samples included patient-caregiver dyads. The patient inclusion criteria were as follows: 18 years of age or older; newly diagnosed with high-grade glioma (grades III and IV) according to the WHO classification; and willing to participate. The patient exclusion criteria were as follows: language barriers; refusal to participate; highly deteriorated health and/or cognition status based on the physician’s opinion; and reticence of the medical staff to propose participation in the study due to the severity of the situation (deterioration of health status, troublesome socio-environmental situation, geographical distance, etc.). The inclusion criteria of the informal caregivers were as follows: 18 years of age or older; most involved person in the patient’s life as defined by the patient; able to speak/read French; and willing to participate. The exclusion criteria of the informal caregivers were as follows: severe cognitive problems based on the physician’s opinion. Written consent forms to participate were collected from every patient and caregiver.

### Data collection

The baseline assessment (T1) was performed 2 to 6 weeks after surgery and before chemo/radiotherapy treatment initiation. For the patient, the following clinical data were gathered using medical records and examination by a senior oncologist/neurologist: type and grade of the glioma; initial WHO performance status; initial treatment plan; and cognitive dysfunction level (defined by a score of less than 24 according to the French version of the mini-mental state exam [20, 21], a widely used tool for the assessment of cognitive failure in clinical practice [22]).

The nature of the relationship between the patient and the informal caregiver was collected (romantic partner, child, or other). The age, gender, educational level, marital status, and number of children were recorded for both the patient and his/her caregiver using self-report questions.

At inclusion (T1) and the 3-month follow-up (T2), quality of life and coping strategies were collected by means of self-reported questionnaires completed by the patients and the caregivers.

- Quality of life was assessed using the French version of the Patient-Generated Index (PGI) [23] for both the patient and the informal caregiver, as well as the French version of the EORTC QLQ-C30 [24] for the patients and the French version of the CareGiver Oncology Quality of Life (CarGOQoL) [25] for the caregivers. The PGI is a well-validated, generic, 15-item questionnaire that assesses the QoL of individuals in the areas most affected by the disease that were previously described as satisfactory for people with cancer [26]. A global index ranges from 0 (lowest QoL) to 100 (highest QoL). The QLQ-C30 version 3 is a well-validated, widely used, specific questionnaire that assesses the QoL of cancer patients, and it includes 30 items that describe five functional scales (physical, role, emotional, cognitive, and social), nine symptom scales, and a global health status scale. The scores for each scale/item range from 0 to 100. We used only the functional scale scores. A high score for a functional scale represents a high/healthy level of functioning. The CarGOQoL is a well-validated specific questionnaire for informal caregivers of cancer patients and includes 29 items describing 10 dimensions: psychological well-being, burden, relationship with health care, administration and finances, coping, physical well-being, self-esteem, leisure time, social support and private life. An index was computed. All dimension scores and the index are on scales of 0–100. A higher score indicates a better QoL.
- Coping strategies were assessed using the Brief Coping Orientation to Problems Experienced Scale (BriefCope) [27]. This questionnaire includes 28 items exploring 14 strategies: self-distraction, active coping, denial, substance use, emotional support use, instrumental support use, behavioral disengagement, venting, positive reframing, planning, humor, acceptance, religion, and self-blame. Confirmatory factor analyses have shown a satisfactory goodness of fit of the French version of the tool [28], encouraging a reduction to 4 dimensions that include social support, problem solving, avoidance, and positive thinking. Scores ranged from 0 to 100. High scores in these 4 dimensions reflect a high tendency to implement the corresponding coping strategies.

### Statistical aspects

After descriptive analyses of the characteristics of patients and caregivers, QoL scores were computed using the algorithms provided by the developers of the tool. The scores of coping were provided in 4 scores. Comparisons between the scores of caregivers and patients (QoL, coping strategies, anxiety, and mood) were performed using the Wilcoxon test (in accordance with the distribution of the variables). To assess the relationships between the coping processes (BriefCope scores) used by the individuals (patients and caregivers) at baseline and QoL scores at the 3-month follow-up, two analyses were performed: i) correlations and multiple comparison corrections (false discovery rate); and ii) actor–partner interdependence model (APIM) to assess the dyadic effects of coping strategies on QoL (PGI scores) based on the hypothesis that the scores within the same dyad are not independent. The APIM was assessed using structural equation modeling [19]. This model is based on the fact that scores within the same dyad are not independent but instead are more similar than the scores of two individuals who are not in the same dyad. The APIM is useful for determining how parameters (QoL and coping strategies) among each participant (namely patients and caregivers) are influenced, not only by internal factors but also by factors related to the other member of the dyad. The APIM produces two actor effects (i.e., each person’s QoL regressed on their own coping strategies) and two partner effects (i.e., each person’s QoL regressed on the other person’s coping strategies). Adjustment for age, sex, and baseline PGI score was performed.

## Results

### Sample

**Fig. 1:**
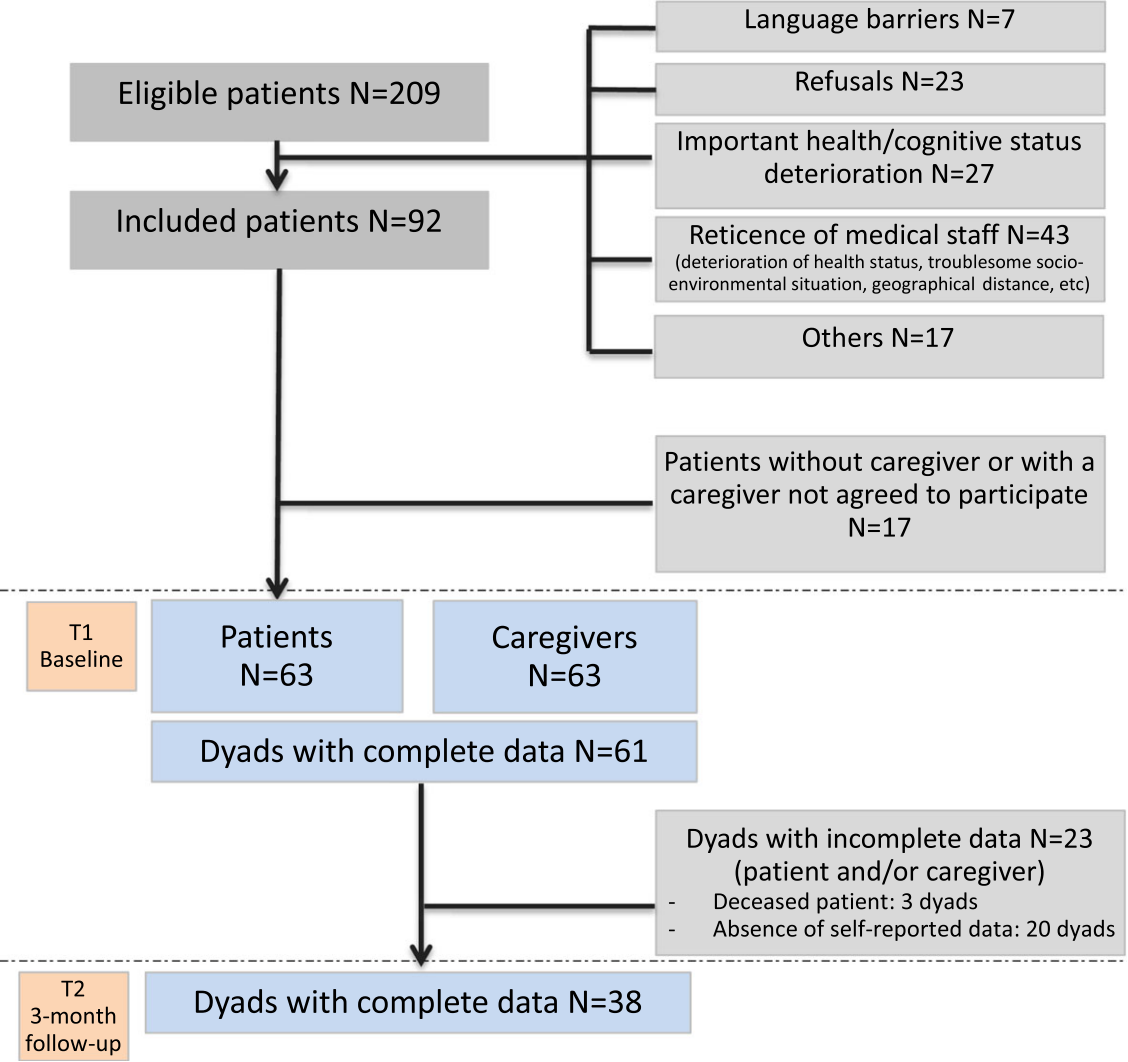
Flow chart

Between April 2014 and May 2016, 209 patients were eligible for inclusion in the cohort. Only 92 patients agreed to participate. The reasons for non-inclusion were as follows: language barriers (7), refusals (23), highly deteriorated health and/or cognition status (27), and reticence of the medical staff (43). The included individuals and the non-included patients were not different in terms of sex, age, or tumor grade. Of the 92 patients, 61 nominated a caregiver who agreed to participate; 61 of the 63 dyads completed the baseline questionnaire. Thirty-eight of the 61 dyads completed the 3-month follow-up. Therefore, the final sample was composed of 38 patient-caregiver dyads. The 38 complete dyads did not differ from the 23 incomplete dyads regarding gender, age, marital status, educational level, tumor grade, and relationship within the dyad. The main characteristics of the dyads are presented in Table 1. A flow chart is presented in Fig. 1.

**Table 1:** Characteristics of the sample

		Patients *N* = 38	
Gender	Women	37%	
Age	Median (IQR)	64 (49–71)	
Marital status	Couple	34	
	Single	4	
Educational level	Low (< 12 y)	17	
	High (> = 12 y)	20	
Days from diagnosis	Median (IQR)	39 (28–62)	
Tumor grade	III	5	
	GBM	33	
First treatment	Biopsy or surgery	30	
	Radiotherapy	34	
	Chemotherapy	38	
		Caregivers *N* = 38	p*
Gender	Women	68%	0.01
Age	Median (IQR)	60 (43–67)	NS
Marital status	Couple	32	NS
	Single	6	
Educational level	Low (< 12 y)	20	NS
	High (> = 12 y)	18	
Relationship with the patient	Romantic partner	30	
	Child	5	
	Friend, family member	3	

* *p*-value for comparsions between patients and caregivers

### Coping strategies of the patients and caregivers

**Fig. 2:**
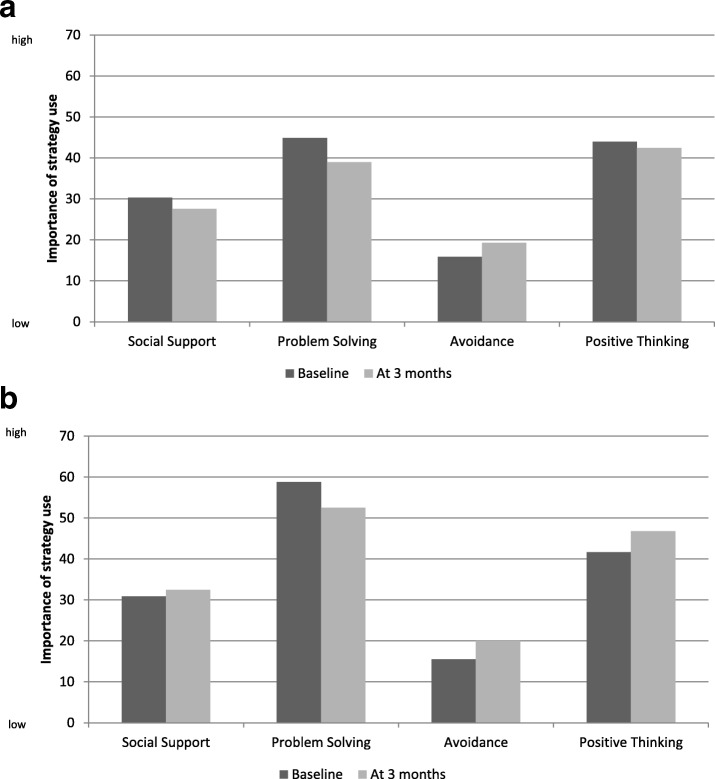
Coping strategies used between baseline and the 3-month follow-up scores range from 0 to 100; High score reflects high implementation of the strategy). **a** Patients: all *p*-value > 0.05 (Wilcoxon paired test). **b** Caregivers: all p-value > 0.05 (Wilcoxon paired test)

Patients used the four types of coping strategies at similar levels at the baseline assessment and at the 3-month follow-up (Fig. 2a). Strategies based on social support and avoidance were the least used and those based on problem-solving were the most used at the two assessments. The strategy most used by the caregiver was problem-solving, both at baseline and the 3-month follow-up. Though the avoidance strategy was the least used, it was used more often at the 3-month follow-up than at the baseline assessment (Fig. 2b). There were no differences between the baseline and 3-month follow-up results for patients and caregivers.

### Relationships between coping strategies at baseline and quality of life at 3 months

The correlations between patient and caregiver scores for coping strategies used at the baseline assessment and the QoL assessment (EORTC QLQ-C30, CarGOQoL, and PGI scores) at the 3-month follow-up are detailed in Table 2. Incidental links were found. The baseline coping strategies was not similarly linked to patients and caregivers QoL at the 3-month assessment. The use of social support was significantly related to lower QoL scores (role functioning and social functioning scores of EORTC QLQ-C30; β = − 0.432 and − 0.485, respectively) for the patients and with higher QoL scores (relationship with health care of CarGOQoL; β =0.350) for the caregivers. For the patient, the use of problem-solving or positive thinking was not related to his/her QoL, while it was related to more satisfactory QoL scores (coping score of CarGOQoL; β = 0.404) for the caregiver. The use of avoidance was linked to a higher QoL score (index PGI, physical function and role functioning dimensions of EORTC QLQ-C30; β from 0.419 to 0.434) for the patients and a lower QoL score (administration and finances, self-esteem, and private life dimensions of CarGOQoL; β from − 0.481 to − 0.415) for the caregivers. We found no correlation between the coping strategies used at the baseline assessment by the patient and the 3-month QoL (PGI index and CarGOQoL scores) of the caregiver, and we found no correlation between the coping strategies used at the baseline by the caregiver and the 3-month QoL (EORTC QLQ-C30 scores) of the patient, except one link: the use of social support by the caregiver at the baseline assessment was associated with a higher 3-month PGI index of the patient (β = 0.583, *p* < 0.001). Expectedly, two coping strategies used by the caregiver at the baseline assessment (problem-solving and positive thinking) were linked to his/her 3-month QoL coping score of CarGOQoL; however no link was found between the use of social support by the caregiver and his/her coping score of CarGOQoL.

**Table 2:** Correlations between coping strategies at baseline and quality of life at 3 months

	Coping strategies of the patient at baseline				Coping strategies of the caregiver at baseline			
	Social Support	Problem Solving	Avoidance	Positive Thinking	Social Support	Problem Solving	Avoidance	Positive Thinking
Patient’s QoL at 3 months								
Index PGI	−0,375	0,037	0,434*	0,013	0,583**	−0,003	0,342	0,053
General Health Status	−0,21	0,021	0,074	0,133	− 0,115	− 0,009	− 0,05	0,125
Physical Functioning	− 0,303	0,086	0,432**	−0,092	− 0,094	0,035	0,183	−0,102
Role Functioning	−0,432**	−0,037	0,419*	0,081	0,107	−0,22	0,113	−0,017
Emotional Functioning	−0,033	−0,018	− 0,167	0,032	0,129	0,31	0,004	0,253
Cognitive Functioning	−0,166	0,197	0,163	−0,131	−0,023	− 0,082	0,301	− 0,073
Social Functioning	−0,485**	− 0,002	0,289	0,002	0,134	−0,161	0,252	0,027
Caregiver’s QoL at 3 months								
Index PGI	0,035	0,212	0,07	0,075	0,112	−0,033	0,039	0,228
Psychological well-being	0,199	0,034	−0,108	−0,067	0,083	0,23	−0,203	0,24
Burden	−0,304	0,19	0,083	0,175	0,075	−0,061	−0,268	0,073
Relationship with health care	0,108	−0,167	−0,192	− 0,219	0,350*	− 0,031	−0,058	− 0,032
Administration and finances	−0,108	0,172	0,202	0,194	−0,056	0,152	−0,415**	−0,1
Coping	0,111	0,053	−0,132	0,211	0,197	0,404*	−0,065	0,512**
Physical well-being	0,022	0,053	0,182	−0,028	0,04	0,049	−0,181	0,126
Self-esteem	0,031	−0,233	−0,018	− 0,025	−0,283	− 0,155	−0,481**	− 0,007
Leisures	0,086	−0,049	−0,024	0,088	0,252	0,174	−0,146	0,134
Social Support	−0,051	−0,019	0,168	0,101	0,209	−0,224	−0,111	− 0,06
Private life	0,053	−0,067	−0,08	0,323	−0,048	0,257	−0,417*	0,061

* *p*-value < 0,05; ** *p*-value < 0.01

Bold values indicate a *p*-value < 0.05

**Fig. 3:**
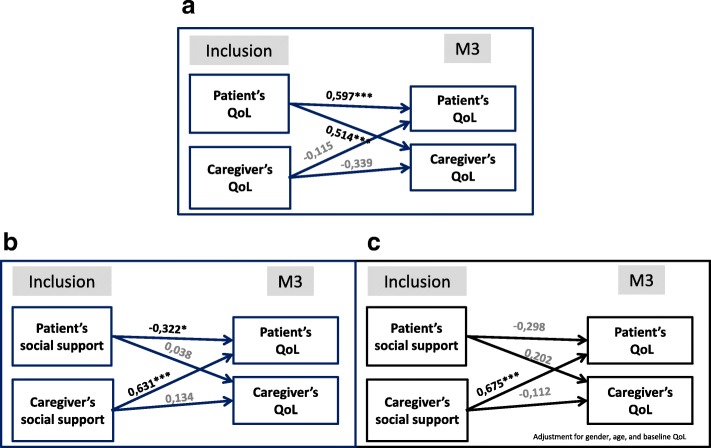
Relations within the patient-caregiver dyad using the actor–partner interdependence model. Numbers are standardized coefficients: β * *p* < .05; ** *p* < .01. **a** Baseline and 3-month QoL (PGI). **b** Baseline coping strategies and the 3-month QoL (PGI) without adjustment. **c** Baseline coping strategies and the 3-month QoL (PGI) after adjustment on age and sex and baseline PGI QoL (patient/caregiver)

Using specific dyadic analyses that integrate the interdependence in two-person relationships, we showed, in Fig. 3, that the level of the patient’s baseline QoL was positively linked with his/her own 3-month QoL and with the 3-month QoL of his/her caregiver (Fig. 3a). The study of the relationships among coping strategies used at the baseline assessment and the 3-month QoL highlighted links between the use of social support (not the use of the 3 other coping strategies) and QoL assessed by the PGI index. Without adjustment, the patient’s 3-month QoL was lower when the patient used the social support strategy at baseline (effect of the use of a coping strategy on their own QoL; negative actor effect; β = − 0.322; *p* = 0.033), but it was higher when his/her caregiver used this strategy (effect of the use of coping strategy on the other member of the dyad; positive partner effect; β = 0.631; *p* < 10^− 3^; Fig. 3b). After adjustment for sex, age, and baseline PGI score, the link between high use of the social support strategy at baseline by the caregiver and the patient’s 3-month QoL, remained present (effect of the use of a coping strategy on the other member of the dyad; positive partner effect; β =0.675; p < 10^− 3^; Fig. 3c). No actor or partner effects were found among the three other coping strategies (positive thinking, problem-solving, and avoidance) used at baseline and the QoL assessed 3 months later (data not illustrated).

## Discussion

The main finding of this study indicates that the QoL of a patient with a newly diagnosed high-grade glioma, assessed away from the diagnosis, may be related not only to the coping strategy he/she mobilizes at the time of diagnosis but also to the coping strategies his/her caregivers mobilize. This observation was already described in cross-sectional studies in both the context of cancer [14, 29, 30] and various non-cancer related contexts [16, 17, 31, 32]. However, to our knowledge, no study has reported this phenomenon across longitudinal designs that nonetheless allow for causality inferences to be made between coping strategies and QoL.

This study found patients and their caregivers implement similar coping strategies. Limited amounts of data are available concerning the coping strategies used by people who are diagnosed with a high-grade glioma [33, 34]. This result suggests that people who know each other very well and who are faced with the same difficult event tend to cope with it similarly. Overall, at the time of the assessment, both mobilized active strategies based on problem-solving and positive thinking more than passive strategies based on looking for social support or avoidance. This trend, already described in the literature in other models of illness [15–17], was always present at the second assessment, 3 months following the diagnosis, during which a sequence of serious events occurs: information about poor prognosis, initiation of the first aggressive treatments, occurrence of serious adverse events due to the treatments, aggravation of disease symptoms, and possible progression of the disease. Because of the rapid progression of the disease, patients with high-grade gliomas and their caregivers only have a short time to adapt. Considering the successive events they endure during this short time, we might expect that individuals develop specific coping strategies over time [35]. However, the individuals did not change the nature of their coping strategies over time, except for the caregivers who mobilized avoidance strategies more often at the second assessment than at the baseline assessment.

This study specifically highlights the distinctive role of the coping strategy based on social support in the self-reported QoL of the ill persons. First, unsurprisingly, the use of a coping strategy based on social support by the patient seemed to be negatively associated with a deterioration of his/her own QoL over time. This result may indicate that a patient who turns to external support to confront the events occurring after diagnosis probably feels badly compared to a patient who does not use social resources, likely due to stronger personal resources that enable him/her to better face the situation alone. To support this assertion, previous studies have described that patient profiles may differ for patients reporting perceived unmet and met needs for supportive care [36, 37].

In contrats, the use of a coping strategy based on social support by the caregiver seemed to positively impact the QoL of the cancer patient assessed away from diagnosis. Because cancer is considered a dyadic stressor, disruptions likewise affect the principal caregiver [38]. We hypothesize that a patient may feel better when he/she realizes that the second member of the dyad implemented social support coping strategies to brave this common, brutal, and tumultuous life event. Knowing that the caregiver is seeking support in this difficult life period may relieve the ill person. The two members of the dyad likely run in opposition “as communicating vessels”: when one of the members feels better, the second member may feel worse. Research has shown the importance of examining dyadic models, that investigatethe ways in which the coping strategies implemented by one member influence outcomes in the other member of the dyad [39]. In this context, the dyadic perspective makes sense.

Because we found that the use of social support strategies may have different impacts on individuals’ QoL depending on the member of the dyad (patient or caregiver), it is important to develop appropriate care services for these persons. Developing a better understanding of the ways in which patients and their relatives support each other and cope together during stressful situations may aid the development of dyad-focused psychological interventions [40, 41]. Future research will benefit from a greater focus on the interactions between patients and their relatives to address the ways a “couple” adapts and copes with a serious disease.

We must note the important role of social support centered on the caregiver. Though the suggestion of psychological support is now established as standard care for the cancer patient, it is time to make similar available to the caregiver. Because everything centers around the patient, it is not always easy for caregivers to receive proper emotional support. An interpretative phenomenological analysis performed on partners of individuals with gliomas showed a relative reluctance of supportive care for various reasons: denial of supportive care need, fear of stigmatization sending back a weaker picture of him/her, and questioning of personal resources and self-esteem [42]. However, talking or sharing experiences with other cancer caregivers was shown as a way of helping them to cope with their situation [43].

### Strengths and limitations

We must mention the representativeness of our sample because of the high proportion of non-included individuals. The neuro-oncologists involved in this project highlighted the difficulty in the assessment of newly diagnosed patients. Patients and caregivers must integrate a large amount of devastating information related to the severity of treatments and the potentially bad evolution of their health status that may occur over a short period of time. We hypothesize that the non-participants probably presented the most severe health and cognitive statuses (including death) were the most disinterested in clinical research issues, and had the most complex family patterns. This statement indicates that our findings may transcribe a partial picture of reality. The comparison of the included individuals and the non-included patients showing that they were not different in terms of sex, age, or tumor grade may be a reassuring element.

Due to the small sample size and low power, moderate associations were possibly missed, and adjustment according to confounding factors was constrained. The small sample size does not allow for a deeper investigation of several associations with QoL or coping, especially investigations regarding sociodemographics (educational level, marital status), disease progression (tumoral progression, metastasis), and the nature of the dyadic relationship. Due to the high variance found among individuals in each group on sociodemographic or clinical variables, we may hypothesize that the results should partially be linked to the specificities of our sample. A larger sample will allow for the confirmation of these findings. However, the specific dyadic analyses, based on APIM that integrates a conceptual view of interdependence in two-person relationships [44, 45], preferentially assesses effects within longitudinal designs, which provides more valid information. The small sample size it is not totally inconsistent with the use of structural equation modeling in the APIM analyses [46].

The delay of the second assessment (3 months after the disease diagnosis) should be considered short. Molassiotis et al. [34] reported that patients usually begin to organize their lives more, accept their limitations, and find ways to manage limitations at least 6 months following disease diagnosis. Reports at the first 3 months following the diagnosis remain interesting.

## Conclusion

The QoL of patients and their natural caregivers three months after the time of diagnosis is directly related to the use of coping strategies based on social support at the time of diagnosis, but is not related to the use of coping strategies based on positive thinking, problem-solving, and avoidance. A better understanding of the ways in which patients and their relatives cope together may aid in the development of personalized, couple-focused psychological interventions.

## Abbreviations

APIM: Actor–Partner Interdependence Model
Brief COPE: Brief Coping Orientation to Problems Experienced
CarGOQoL: CareGiver Oncology Quality of Life
EORTC: European Organisation for Research and Treatment of Cancer
PGI: Patient-Generated Index
QLQ-C30: Quality of Life Questionnaire – Core 30
QoL: Quality of Life
SIRIC: Site de Recherche Intégrée sur le Cancer
